# The relationship between script concordance test scores in an obste rics-gynecology rotation and global performance assessments in the curriculum

**DOI:** 10.5116/ijme.4d21.bf89

**Published:** 2011-01-07

**Authors:** Patricia Monnier, Marie-Josee Bedard, Robert Gagnon, Bernard Charlin

**Affiliations:** 1 MUHC Reproductive Center, Royal Victoria Hospital, McGill University, Canada; 2 Department of Obstetrics and Gynecology, Centre Hospitalier de lUniversite de Montreal, Universite de Montreal, Canada; 3 Unit for Research and Development in Health Sciences Education, Universite de Montreal, Canada

**Keywords:** Clerkship, dean’s letter, in-training evaluation reports, key features examinations, script concordance test

## Abstract

Objectives: To determine the relationship between script concordance test scores obtained at the end of clerkship rotation in ob-gyn and global performance measures for the entire curriculum as expressed by a preclinical and clerkship score in the Dean’s Letter. The relationship also considered two other existing instruments.

**Methods:**

A cross-sectional study was carried out on a convenience sample of 129 clerkship students. Three instruments (Script Concordance Test, Key-Features Examinations and In-Training Report) were used to assess clinical reasoning. Data were collected from four Montreal University Hospitals at the end of four consecutive obstetrics and gynecology rotations. The data pertaining to the Dean’s Letter were collected at the end of the clerkship training period.

**Results:**

Cronbach’s alpha values were 0.67 for the script concordance test and 0.36 for Key Features Examinations. A significantly positive correlation was found between the preclinical (r = 0.260, p = 0.01) and clerkship (r = 0.232, p = 0.01) scores of the Dean's Letter and the script concordance test. Regression analysis showed that the best predictor for the clerkship score of the Dean’s Letter was the script concordance test (r = 0.226, p = 0.014).

**Conclusions:**

The script concordance test was associated with the scores in the Dean’s Letter in comparison with two other scales, which suggests that the test can be a useful tool for clinical educators who are engaged in the assessment of clinical reasoning, particularly in clerkship students. However, further work is required to establish this association.

## Introduction

Evaluation of clinical reasoning presents a particular challenge to educators. Whereas factual knowledge is relatively straightforward to assess using traditional testing methods (such as multiple choice or short-answer examinations), standardized measures of clinical reasoning are lacking. At present, assessment of clinical reasoning in clerkships involves global ratings by staff who observe students in the clinical setting over the length of a clinical rotation.^1^ Key features exams represent another commonly used instrument. Based on the concept of critical steps or “key features”, it tests clinical decision-making skills in written or computer-based formats.^2^The script concordance test (SCT) is an innovative tool that assesses clinical data interpretation (CDI), a crucial component of clinical reasoning.^3^ SCT assesses how examinees actively process information to confirm or eliminate diagnostic and management options with a series of qualitative judgments.^4^ Students, residents, and physicians thoroughly accept the SCT format and find it relevant and interesting to complete. Its validity is increasingly documented.^3^ The goal of this study is to verify whether the SCT would prove to be a useful adjunct for clinical reasoning assessment.In many North American medical schools, all marks obtained during the curriculum are averaged in a “Dean’s Letter” by the two preclinical and clerkship scores. This Dean’s Letter represents an assessment summary of students’ performance during the preclinical and clerkship parts of the curriculum. As such, it is widely used for selection in residency programs.^5^The study examines the link between SCT scores obtained during clerkship rotation in obstetrics and gynecology (ob-gyn) and global performance measures mentioned in the Dean’s Letter. The hypothesis was that, because CDI is related both to knowledge organization and clinical reasoning, SCT results would correlate positively with the two scores of the Dean’s Letter. We also compared this new instrument with the two existing measures of clinical reasoning in the ob-gyn clerkship rotation of our institution: Key Features Examination (KFE) and In-Training Evaluation Reports (IT/CR).

## Methods

### Measures

**SCT**- A 27-case, 82-question SCT in ob-gyn was developed for the study, in accordance with published guidelines.^6^ It was designed to measure CDI in a series of clinical situations representative of ob-gyn practice and clerkship educational objectives. Situations (cases) were depicted in short vignettes, and three or four related questions were asked. Each question was made up of three parts: the first part (“If you were thinking of”) included a diagnostic hypothesis, an investigative action or a treatment option; the second part (“and then you find”) offered additional information, such as clinical, imaging or laboratory test data; the third part (“this hypothesis becomes”) was a five-point Likert-type scale that captured the student’s decision (See Table 1). The examinee’s response to each question was compared with responses from the experts’ panel. Credit was assigned to each response based on the number of experts choosing that response. A maximum score of 1 was given for the response chosen by most experts (i.e., the modal response). Other responses were given partial credit, according to the number of experts choosing them. Responses that were not selected by experts received a zero score. The sum of credits obtained on each item determined the total score for the test.

**Table 1:** Example of an SCT case and questions

The clinical vignette: A 17-year old woman presented with primary amenorrhoea. Her family history is unremarkable. Her mother had a normal vaginal delivery after an uneventful pregnancy. She practices karate twice a week and has a good relationship with her parents.			
	If you were thinking of ...	… and then you find ...	this hypothesis becomes
Q1	Chromosomal abnormality	A pelvic ultrasound shows that the uterus is present	- 2 very unlikely;
			- 1 less likely;
			0 neither likely nor unlikely;
			+ 1 more likely;
			+ 2 very likely
Q2	Androgen insensitivity syndrome formerly known as testicular feminisation	Examination reveals bilateral galactorrhoea	- 2 very unlikely;
			- 1 less likely;
			0 neither likely nor unlikely;
			+ 1 more likely;
			+ 2 very likely
Q3	A craniopharyngioma	Elevated serum FSH levels	- 2 very unlikely;
			- 1 less likely;
			0 neither likely nor unlikely;
			+ 1 more likely;
			+ 2 very likely

**KFE** -The examination, held at the end of the ob-gyn clerkship rotation, comprised 12 cases with key-feature questions taken from a pool of 50 cases developed by ob-gyn faculty members. Cases and questions had been developed according to the methodology described by Farmer and Page.^2^ They were correlated with the rotation’s educational objectives. All questions were distributed evenly between obstetrics and gynecology. Students had 90 minutes to complete the exam. Scores were in percentage points.**IT/CRs** -At the end of each rotation, supervisors evaluated students’ competencies based on an in-training evaluation report and CanMEDS roles.^7^ From these reports, we extracted data on items related to clinical reasoning: clinical knowledge, formulation of clinical problems, development of relevant diagnostic hypotheses, formulation and rationale for proposed investigations and treatment. The IT/CR score was the mean of scores obtained on these 4 items, expressed on a 1 to 10 scale.The Dean’s Letter-as pointed out earlier, is a summary of all evaluations obtained by medical students during their curriculum. It is made up of two scores: preclinical and clerkship. For the latter, the marks are based on 70% of the in-training evaluation reports and on 30% of end-of-rotation exams. In these rotations, exams are a combination of the KFE, multiple-choice questions, and the Objective Structured Clinical Examination (OSCE).

### Design

The study design was cross-sectional. Data on SCT, KFE and clinical reasoning ratings from IT/CR were collected at the end of four consecutive 8-week ob-gyn rotations in the Université de Montréal’s four teaching hospitals. In ob-gyn, the OSCE is used to assess data collection techniques and strategies rather than clinical reasoning, and multiple choice questions are used to assess factual knowledge. Data from these two tools were therefore not included in the study. Data on the Dean’s Letter were collected at the end of the clerkship. The study received approval from the University’s Institutional Review Board.

### Participants

One hundred and fifty-four (154) clerkship students i.e., 90% of the 171 who were in these four consecutive rotations, signed an informed consent to complete the SCT. One hundred and twenty-nine (129) of them (75.4% of the 171) agreed to provide their identification number, thus allowing comparison of the results on the various tools (SCT, KFE, IT/CR, Dean’s Letter). To build the SCT answer key, all members of the ob-gyn teaching staff of the institution were invited to complete the test on line. Recruitment was stopped when fifteen (15) teachers had signed up. Anonymity was assured.

### Statistical analysis

Distribution normality was evaluated using the Kolmogorov-Smirnov statistical test. SCT optimization was achieved by excluding questions with item-total correlations lower than 0.05.^4^ Score reliability for SCT and KFE was tested using Cronbach’s alpha coefficient. The relationship between the preclinical and the clerkship scores of the Dean’s Letter, and the three other tools was assessed with Pearson’s correlation coefficient. Multiple linear regression analysis was used to identify the relative contribution of each measure for predicting the clerkship score of the Dean’s Letter as an outcome variable. All p-values were considered significant at at ≤ 0.05. This analysis was done with Statistical Package for Social Sciences (SPSS) software, version 16.0.

## Results

One hundred and fifty-four (154) clerkship students sat for the paper and pencil 82-item SCT. Of those, 70% were female and 30% were male, which is comparable with the proportion in our faculty of medicine. Students completed the test in 59 minutes (± 18), and experts in 42 minutes (± 8). When compared, scores from the 129 students who agreed to provide their identity and the ones from the 25 who refused did not show any significant differences (p=0.89). The optimization process of eliminating items with item/total negative correlation resulted in the removal of 25 questions. As a result, the SCT comprised 26 vignettes (13 in obstetrics and 13 in gynecology) and 57 questions. Our analyses were based on the optimized SCT scores. The Cronbach alpha coefficient value for the optimized SCT was 0.67; value for the KFE was 0.36. The mean score and standard deviation of data obtained for the five assessment tools were as follows: optimized SCT 36.8 (4.8); IT/CR 8.4 (0.4); KFE 69.9 (10.9); preclinical Dean’s Letter 3.4 (0.4); clerkship Dean’s Letter 3.3 (0.2).

Pearson correlations between the assessment tools are presented in Table 2. All tools were positively correlated with the preclinical score of the Dean’s Letter. Only SCT was positively correlated with the clerkship score of the Dean’s Letter, with a p-value of 0.01. SCT is positively correlated with all tools, with a p-value of 0.01, excepted with KFE. The latter is positively correlated exclusively with the preclinical Dean’s Letter score (0.181; p = 0.05). Table 3 presents the results of regression analysis. Among tools examined, the SCT is the best and only significant predictor of clerkship Dean’s Letter score. The multiple regression coefficient is 0.226.

**Table 2:** Pearson Correlations between the assessment tools described in the study among 129 clerkship students at Université de Montréal; IT/CR, Clinical reasoning items within in-training evaluation report; KFE, Key features examination; SCT, Script Concordance Test

	Dean’s Letter Preclinical score	Dean’s Letter Clerkship score	IT/CR	KFE
Preclinical Dean’s Letter				
Clerkship Dean’s Letter	0.530^✝^			
IT/CR	0.237^✝^	0.102		
KFE	0.181*	0.113	-0.005	
SCT	0.260^✝^	0.232^✝^	0.262^✝^	0.052

* Correlation is significant at the 0.05 level

^✝^ Correlation is significant at the 0.01 level

**Table 3:** The outcome variable is the clerkship score of the Dean’s Letter; data on 129 clerkship students at the University of Montreal; IT/CR, Clinical reasoning items within in-training evaluation report; KFE, Key features examination; SCT, Script Concordance Test

Model	Unstandardized coefficients		Standardized coefficients	t	P
	B	SE*	Beta		
Constant	2.630	0.384		6.845	0.000
KFE	0.002	0.002	0.078	0.893	0.374
IT/CR	0.024	0.045	0.049	0.544	0.588
SCT	0.010	0.004	0.226	2.485	0.014

* Standard error

## Discussion

We made the hypothesis that, as SCT probes data interpretation, a crucial task of the clinical reasoning process that requires organized knowledge, its results would correlate positively with the two scores of the Dean’s Letter, clerkship and preclinical. Data confirm this hypothesis. It is important to point out that SCT scores applied to ob-gyn rotations only, while the scores of the Dean’s Letter’s applied to the entire medical curriculum. These results are in line with Brailovsky’s findings,^8^ which showed that SCT scores in a specific field (surgery) at the end of clerkship predicted scores obtained on measures of clinical reasoning performance two years later, at the end of the family medicine residency. Brailovsky’s conclusion was that students who showed good organization of clinical knowledge at an early stage in training were expected to show good organizational skills in similar evaluations later on.The ob-gyn SCT showed acceptable reliability (Cronbach alpha value at 0.67) for an in-training exam requiring about one hour of testing and it was positively correlated with clinical reasoning ratings taken from ob-gyn IT/CR. We were surprised by the low psychometric properties of the ob-gyn KFE (alpha value was at 0.36). This test was correlated only with the preclinical part of the Dean’s Letter, thus being linked to a greater extent with factual than clinical knowledge. A misconstruing of this specific test could explain the situation. All KFEs used in clerkship rotations are currently being reviewed in our institution.

The present study has certain limitations that need to be taken into account. They concern clerkship students who went through 4 consecutive ob-gyn rotations. We do not have data on the 10% who did not agree to take the SCT. Among those who participated, some did not agree to provide access to their other curriculum data. We therefore have data on only 75.4% of the cohort. Study results will have to be reproduced in other institutions with their own panels and examinees. Another limitation is the low reliability of our ob-gyn KFE. The lack of correlation of this measure with the other measures may be the result of this low reliability. The make-up of the panel is another significant issue. Our panel of 15 included experts who were sometimes specialized in one specific ob-gyn area (high-risk pregnancies, oncology, gynecology, infertility). In the future, it will be interesting to examine if answer keys developed by sub-specialists improved students’ score reliability.

The study was designed to explore the role SCTs may have on clinical reasoning assessment within the curriculum. Correlation and regression analyses showed significant associations between SCTs and both the preclinical and clerkship scores of the Dean’s Letter. These associations,

stronger than those of existing tools used to assess clinical reasoning, suggest the usefulness of SCTs in assessment strategies. However, further work and reproduction of the findings in other settings will be required to establish this association.
